# Effects of Stimulus Type and Strategy on Mental Rotation Network: An Activation Likelihood Estimation Meta-Analysis

**DOI:** 10.3389/fnhum.2015.00693

**Published:** 2016-01-07

**Authors:** Barbara Tomasino, Michele Gremese

**Affiliations:** IRCCS “E. Medea,”San Vito al Tagliamento, Italy

**Keywords:** ALE meta-analysis, mental rotation, mental imagery, fMRI

## Abstract

We can predict how an object would look like if we were to see it from different viewpoints. The brain network governing mental rotation (MR) has been studied using a variety of stimuli and tasks instructions. By using activation likelihood estimation (ALE) meta-analysis we tested whether different MR networks can be modulated by the type of stimulus (body vs. non-body parts) or by the type of tasks instructions (motor imagery-based vs. non-motor imagery-based MR instructions). Testing for the bodily and non-bodily stimulus axis revealed a bilateral sensorimotor activation for bodily-related as compared to non-bodily-related stimuli and a posterior right lateralized activation for non-bodily-related as compared to bodily-related stimuli. A top-down modulation of the network was exerted by the MR tasks instructions with a bilateral (preferentially sensorimotor left) network for motor imagery- vs. non-motor imagery-based MR instructions and the latter activating a preferentially posterior right occipito-temporal-parietal network. The present quantitative meta-analysis summarizes and amends previous descriptions of the brain network related to MR and shows how it is modulated by top-down and bottom-up experimental factors.

## Introduction

Imagining scenes, sounds and actions, in the absence of appropriate stimuli for the relevant perception, takes place through mental imagery (Kosslyn et al., 1995, 2001). These images can also be combined and modified in novel ways. Mental rotation (hereafter MR) occurs when thinking how an object would look like if seen from a different viewpoint (Shepard and Metzler, 1971; Corballis, 1997).

Processes involved in MR have been studied extensively since Shepard and Metzler (1971) asked participants to decide whether two differently oriented three-dimensional objects were either identical, or mirror images of each other. A proportional relationship was found between the angle of rotation and the time people needed to make a decision. These results suggest that subjects form a visual image of an object and rotate this image until it is congruent with the target stimulus. This pattern has been found with three-dimensional pictures (i.e., 3D cubes), alphanumeric characters (Corballis and Sergent, 1989), abstract pictures, and body parts such as hands (Parsons, 1987; Parsons et al., 1995, 1998; Parsons and Fox, 1998). In addition, RTs for MR of body parts reflect the degree of awkwardness of the picture orientation (Parsons, 1987), because subjects imagine a spatial transformation of their own body part and report kinesthetic sensations (Parsons, 1987).

MR is a complex cognitive task, involving different sub-processes such as object orientation discrimination, visual imagery, mental representation of a stimulus, dynamic spatial transformation of this image, mental comparison, attentional and working memory stages, decision-making and implementation of this decision into a motor output (Kosslyn et al., 1995; Wexler et al., 1998).

Different factors can influence MR operations. In the present study we focused on the effect of the type of stimulus and the effect of instructions which may trigger a specific MR strategy^1^. With stimulus we mean the type of picture presented on the screen. With strategy we mean the instructions guiding the participants in solving the task. The strategy can be motor imagery-based or visual-imagery based instructions (see in the Method section below some examples). In Table 1 we evidenced in the column “instruction” the strategy given by the experimenters. For instance, all the studies in which the instructions explicitly required participants to imagine hand movements (e.g., “by imagining rotating their own hand into the position of the hand presented”; “simulating a motor rotation of one's own hand”; “MR as a consequence of their hand rotational movement”; see for instance in Vingerhoets et al.'s study (2002): “participants imagined moving both their hands in the hand condition, while imagining manipulating objects with their hand of preference (right hand) in the tool condition”) were included under the category “motor strategy.” All the studies in which the instructions explicitly required participants to imagine the stimulus rotating in the space (e.g., “as a consequence of an external force rotating the object”; see for instance in Barnes et al.'s study (2000): “ […] In the target phase one of the figures was offset and subjects were told to visualize it rotating in a continuous movement until it aligned with the other figure, and then to decide whether the two figures were identical or mirror images of each other”; or in: Keehner et al.'s (2006): ”[…] imagined that the table rotated while they remained stationary”) were included under the category “visual strategy.”

**Table 1:** **Studies' details included in the meta-analyses**.

**Study N°**	**Authors**	**Stimuli**	**Instructions**	**Paradigm**	**Only MOTOR**	**Only Egocentric**	**Scan**	**RM**	**Subjects**	**M/F**	**Handedness**	**Coord**.	**Analysis**	**Contrast**	**Foci**
1	Alivisatos and Petrides, 1997	Alphanumeric pair	Same or reversed				PET	−	10	10M	R	Talairach	Pixar 3D; non-spec	MR>control	9
2	Aso et al., 2007	Alphanumeric pair	Same or reversed				MRI	3T	12	12M	R	Talairach	spm2	MR>control	5
3	Barnes et al., 2000	3D cubes pair	Look at stimuli pair: identical or mirror? In the target phase one of the figures was offset and subjects were told to visualize it rotating in a continuous movement until it aligned with the other figure, and then to decide whether the two figures were identical or mirror images of each other	VISUAL			MRI	1.5T	6	4M;2F	Not reported	Talairach	Not spec	MR>control	6
4	Blanke et al., 2010	Body	“participants were asked to make right-left judgments of the schematic human figure after having imagined themselves to be in the figure's body position”	MOTOR/EGOCENTRIC		E	MRI	1.5T	14	7M;7F	13R;1L	MNI	spm2	MR>control	7
5	Bonda et al., 1995	Hands	MR of subjects' hands, left right decisions A questionnaire was administered to each subject at the end of the test. The responses revealed a certain variability in the strategies used by the different subjects. All strategies, however, involved reference to the subject's body by requiring mental rotation of his hand in order to match the orientation of the stimulus shown	MOTOR	M		PET	1.5T	16	16M	R	Talairach	Other	MR>control	21
6	Butler et al., 2004	3D cubes pair	Same different “subject were instructed to mentally rotate the figures into alignment in order to decide if they were the same or different”	VISUAL			MRI	3T	25	12M; 13F	R	Talairach	spm99	MR male>control	21
7	Corradi-Dell'Acqua et al., 2009	Hands	Handedness decision for hands “participants were asked to accomplish the task using different strategies, that is, either by imagining the arm stimulus rotating until this could be wedged in the human photograph (visual strategy) or by ignoring the human photograph and imagining to rotate their own arm until this reached the position depicted in the screen (motor strategy)”	MOTOR	M		MRI	3T	17	17M	R	MNI	spm5	MR hands>control	1
8	Creem et al., 2001	An array of four objects	Imagine transformation of one's body. Subjects updated the position of one of four external objects from memory after they had performed an imagined self-rotation to a new position	MOTOR EGOCENTRIC		E	MRI	1.5T	12	6M;6F	R	Talairach	AFNI	MR>control	15
9	Creem-Regehr et al., 2007	Hand in the center of 6 spheres	Hand: “instructed to decide whether the hand presented was a right or left hand by imagining rotating their own hand into the position of the hand presented”	Motor/Egocentric	M		MRI	1.5T	18	7M	R	MNI	SPM99	Hand>control	15
9	Creem-Regehr et al., 2007	Hand in the center of 6 spheres	Viewer: “instructed to imagine that they were standing at the blue sphere, and from that new imagined perspective to decide whether the previously named hand part, “thumb” or “pinky,” was on their right or left”	Motor/Egocentric		E	MRI	1.5T	18	7M	R	MNI	SPM99	Viewer>control	9
10	de Lange et al., 2005	Hands	Hand laterality task We used two tasks, an motor imagery (MI) and a visual imagery (VI) task. Four line drawings of hands (left or right hand, viewed either from the back or from the palm) served as stimuli for the MI task. Four typographical characters (F, G, J, and R, in Times New Roman font) served as stimuli for the VI task	MOTOR and visual			MRI	1.5T	6	6M	R	Talairach	spm99	MR>control	10
	de Lange et al., 2005			MOTOR	M									hands>control	7
11	de Lange et al., 2006	Hands in the center of six spheres	Handedness decision	MOTOR	M		MRI	3T	17	16M	R	Talairach	spm2	MR>control	6
12	Ferri et al., 2012	Hands	Handedness decision In the current fMRI study, we tested this hypothesis by making participants undergo a hand laterality judgment task, which is known to be solved by simulating a motor rotation of one′s own hand	MOTOR	M		MRI	3T	18	9M;9F	R	MNI	spm8	MR>control	9
13	Gogos et al., 2010	Alphanumeric characters	Correct or mirror orientation				MRI	3T	9	9F	R	Talairach	spm5	MR>control	11
14	Harris et al., 2000	Alphanumeric characters pair	Same mirror decisions				PET	-	7	4M; 3F	R	Talairach	ANALYZE; spm 96	MR>control	1
15	Halari et al., 2006	3D shapes pair	Same-mirror decisions				MRI	1.5T	19	9M;10F	R	MNI	spm99	MR male>fixation	11
	Halari et al., 2006													MR female>fixation	4
16	Hugdahl et al., 2006	3D cubes pair	Same different decisions				MRI	1.5T	11	6M;5F	R	Talairach	spm96	MR general>control	4
17	Johnston et al., 2004	Abstract novel forms pair	Visuo-spatial normalization Same-mirror decisions	VISUAL			MRI	1.5T	9	5M;4F	R	MNI	spm99	MR>control	3
18	Jordan et al., 2001	3D Abstract shape Letters pair	Same mirror decisions on pair of stimuli				MRI	1.5T	9	1M; 8F	R	MNI	spm99	MR cube> fixation	6
	Jordan et al., 2001													MR abstract> fixation	7
	Jordan et al., 2001													MR alphanumerics>fixation	5
19	Jordan et al., 2002	3D Letter Abstract pair	Same-different judgment “Subjects were told to turn the right figure clockwise to match the left, in order to decide whether it is the same or the mirror image	VISUAL			MRI	1.5T	24	10M; 14F	1L	MNI	spm99	MR male>control	6
	Jordan et al., 2002													MR female>control	13
20	Kawamichi et al., 2007a	3D cubes but 2D and 3D pair	“Only 3D rotation implicitly requires subjects to construct and manipulate 3D images with visualizations of the hidden parts; this plays an important role in visuomotor tasks such as preshaping This implies that task difficulty enhanced by rotation dimensionality is a major factor related to the selection of motor strategy” Same mirror decision	MOTOR (3D rotation) and VISUAL (2D rotations)	M		MRI	1.5T	14	14M	R	MNI	spm99	MR big rotation cube>fixation	11
	Kawamichi et al., 2007a				M									MR small rotation cube>fixation	9
	Kawamichi et al., 2007a			VISUAL										MR big rotation 2D>fixation	10
	Kawamichi et al., 2007a			VISUAL										MR small rotation 2D>fixation	10
21	Keehner et al., 2006	A circular table with a ball on top	Imagined that the table rotated while they remained stationary	VISUAL			MRI	1.5T	14	7M; 7F	R	MNI	spm2	MR>control	4
22	Kosslyn et al., 1998	Hands 3 D Cubes pair	Same mirror decisions Mechanisms that prepare motor movements and mechanisms that do not	MOTOR and non-MOTOR			PET	-	20	20M	R	Talairach	spm95	MR objects>control	8
	Kosslyn et al., 1998			MOTOR	M									MR hands>control	9
23	Kosslyn et al., 2001	Cubes pair	Internal strategy (as a consequence of their hand rotational movement)	MOTOR	M		PET	-	8	8M	R	Talairach	spm95	MR cube motor>control	9
	Kosslyn et al., 2001		Or external strategy (as a consequence of an external force rotating the object)	VISUAL										MR cube visual>control	7
24	Kucian et al., 2007	2D stimuli pair	Same-mirror decisions				MRI	1.5T	20	10M;10F	R	Talairach	spm99	MR>fixation	13
25	Lambrey et al., 2012	Table plus avatar	Self-rotation (taking a new perspective at a different position) AND Array rotation table rotation to a new perspective)	MOTOR/EGOCENTRIC AND VISUAL			MRI	3T	18	9M;9F	R	MNI	spm5	MR general>control	23
	Lambrey et al., 2012			MOTOR/EGOCENTRIC		E								MR body>fixation	20
	Lambrey et al., 2012			VISUAL										MR cube>control	33
26	Lamm et al., 2001	3D cubes pair	Same different decisions				MRI	3T	13	13M	R	MNI	spm99	MR>control	11
27	Lamm et al., 2007	2D geometrical figures pair	“subjects were instructed to rotate the figure until this position was reached as this would allow them to directly compare it to the matching figure”	VISUAL			MRI	3T	13	13M	R	Talairach	spm2	MR>control	4
28	Levin et al., 2005	3D Cubes pair	Same-DIFFERENT decision				MRI	1.5T	49 MR; 12WM	24M; 6M	1L	Talairach	spm	MR>control 1	4
29	Logie et al., 2011	3D Cubes pair	Same-mirror decision				MRI	1.5T	21	7M; 14F	R	MNI	spm5	MR>control	2
30	Milivojevic et al., 2009	Alphanumeric	Normal-mirror decision				MRI	1.5T	14	8M;6F	R	MNI	spm5	MR>control	10
31	Ng et al., 2001	Alphanumeric pair	Same different decisions “Subjects were asked mentally to rotate the bottom L to the same side of the square as the top L was situated and to determine whether the configuration of the two L are the same”	VISUAL			MRI	1.5T	8	8M	R	Talairach	other	MR>control	9
32	Papeo et al., 2012	Hands 3D cubes	Handedness decision And visuo-spatial In the motor strategy based mental rotation task, participants were instructed to decide whether each photograph depicted a left hand or a right hand, by imaging moving their own hands until it reached the position of the hand stimulus on the screen (motor strategy). In the visuospatial strategy-based mental rotation task, participants decided whether a red marker on either arm of the 3-D object was at the left or right of the screen midline, after having mentally visualized the object rotating and aligning with the midsagittal line of the computer screen (visuospatial strategy)	VISUAL AND MOTOR STRATEGY			MRI	3T	18	18 F	R	MNI	spm5	MR general>control	15
	Papeo et al., 2012			MOTOR	M									MR hands>control	6
	Papeo et al., 2012			VISUAL										MR cube>control	6
33	Parsons et al., 1995	Hands	MR of subjects' hands, left right decisions viewers solve this visual shape task by mentally modeling it as a reaching task implicitly moving their left hand into the orientation of any left-hand stimulus (and conversely for a right-hand stimulus)	MOTOR	M		PET	-	7	6M;1F	R	Talairach	other	MR left hand>Fixation	28
	Parsons et al., 1995				M									MR right hand>fixation	32
34	Paschke et al., 2012	3D cubes pair	Same-mirror decision				MRI	3T	10	10M	8R;2L	MNI	spm8	MR>control	20
35	Podzebenko et al., 2002	Alphanumeric	normal-mirror decision				MRI	1.5T	10	5M;5F	7R;3L	Talairach	MEDx-SPM99	MR>control	15
36	Podzebenko et al., 2005	Alphanumeric	Normal or mirror “mentally rotate the stimulus to the near upright position and to indicate their orientation decision”	VISUAL			MRI	1.5T	16	8M;8F	13R;3L	MNI	spm99	MR>rest	12
37	Schendan and Stern, 2007	3D cubes pair	Same-mirror decisions				MRI	3T	13	6M;7F	R	MNI	spm99	MR>control	26
38	Schöning et al., 2007	3D cubes pair	Same-mirror decisions				MRI	3T	24	12M;12F	R	MNI	spm2	MR>control	30
39	Seurinck et al., 2004	Hands Tools pair	MR of hands and hands-related objects known to evoke egocentric motor strategy Same mirror judjments during egocentric mental rotation of handsand tools	Motor strategy	M		MRI	1.5T	22	11M; 11F	R	Talairach	spm99	MR hands male>control	8
	Seurinck et al., 2004				M									MR hands female>control	10
	Seurinck et al., 2004				M									MR objects male>control	14
	Seurinck et al., 2004				M									MR objects female>control	15
40	Seurinck et al., 2005	Hands Tools pair	Same mirror judjments	Motor strategy	M		MRI	1.5T	24	24F	12R;12L	Talairach	spm99	MR hands>control	14
	Seurinck et al., 2005			Motor strategy	M									MR objects>control	10
41	Seurinck et al., 2011	Alphanumeric	Normal-mirror decision				MRI	3T	16	16M	R	MNI??	spm5	MR>control	16
42	Sluming et al., 2007	3D cubes pair	Same mirror judjments				MRI	1.5T	20	20M	R	MNI	spm99	MR>control	14
43	Stoodley et al., 2012	Alphanumeric	Normal-mirror decision				MRI	3T	9	9M	R	MNI	spm8	MR>control	18
44	Suchan et al., 2002	2D abstract	“decide whether the right matrix was an exact 90° of the left”	VISUAL			PET	-	10	4M; 6F	R	Talairach	not spec	MR>control	14
45	Suchan et al., 2006	2D abstract 3D cubes pair	“Subjects were asked to compare or rotate the stimuli either to the left or the right and indicate whether the stimuli are identical or whether the stimulus presented on the right was an exact 90° rotation of the left or first stimulus.” Same-different decisions	VISUAL			MRI	1.5T	11	6M; 5F	R	Talairach	spm99	MR cube>control	3
	Suchan et al., 2006													MR abstract 1>control	12
	Suchan et al., 2006													MR abstract2>control	4
46	Thomsen et al., 2000	3D cubes pair	Same different				MRI	1.5T	11	6M;5F	R	Talairach	spm96	MR general>control	4
	Thomsen et al., 2000	3D cubes pair	Same different				MRI	1.5T	11	6M;5F	R	Talairach	spm96	MR cube>control	4
47	Vanrie et al., 2002	3D shapes pair	Same different				MRI	1.5T	6	3M;3F	R	MNI	spm96	MR abstract 1>control	6
	Vanrie et al., 2002													MR abstract 2>control	8
48	Vingerhoets et al., 2001	Alphanumeric	Normal or backward?				PET	-	10	5M; 5F	R	MNI	spm96	MR>control	1
	Vingerhoets et al., 2001	Alphanumeric	Normal or backward?				PET	-	10	5M; 5F	R	MNI	spm96	alphanumeric>control	1
49	Vingerhoets et al., 2002	Hands Tools pair	With hands Tools Same different decisions “*participants imagined moving both their hands in the hand condition, while imagining manipulating objects with their hand of preference (right hand) in the tool condition*”	MOTOR	M		MRI	1.5T	12	12M	R	Talairach	spm99	MR hands>control	6
				MOTOR	M									MR objects>control	9
50	Wartenburger et al., 2009	2D abstract pair	Identical or rotated decisions “participants were explicitly instructed to mentally mirror the two dimensional figures”	VISUAL			MRI	1.5T	15	15M	R	MNI	FSL	MR>control	5
51	Weiss et al., 2003	3D cube pair	Same different decisions				MRI	1.5T	20	10M;10F	R	Talairach	spm99	MR cube>control	7
52	Weiss et al., 2009	Alphanumeric	Canonical or mirror decision				MRI	3T	16	16M	R	MNI	spm2	MR general>rest	24
53	Wilson and Farah, 2006	Objects Alphanumeric	In object MR task decided “whether a dot was on the left or the right side of the object” In the letter MR: normal or reversal decision	VISUAL			MRI	1.5T	7	4M;3F	R	MNI	spm99	MR>fixation	7
				VISUAL										MR_object>fixation	4
54	Wolbers et al., 2003	3D cubes	Mental rotation combined with motor imagery of hands In order to manipulate the rotation strategies, the two experimental sessions required participants to imagine themselves grasping the object with their right (active_right) or left (active_left) hand. Following the disappearance of the figure, a blank screen was shown for 2000 ms, then a different stimulus was presented. The subject was now required mentally to rotate the first stimulus along the indicated axis to determine whether or not both were identical	MOTOR	M		MRI	1.5T	13	5M;8F	R	MNI	SPM99	MR_right>control	3
	Wolbers et al., 2003			MOTOR	M									MR_left>control	3
55	Wraga et al., 2003	Hands 3D cubes pair	Same different decisions Hands-objects blocks (MOTOR)	MOTOR	M		PET	-	16	16M	R	Talairach	spm95	MR hands>control	5
			Objects-objects blocks (VISUAL)	VISUAL										MR objects>control	6
56	Wraga et al., 2005	3D cube plus cue	OBJECT “participants imagined rotating the object so that one of its ends was aligned with the prompt”	Visual			MRI	1.5T	11	7M;4F	R	Talairach	spm99	MR cube>fixation	7
		3D cube plus cue	SELF “imagined rotating themselves to the location of the T-prompt”	Motor/EGOCENTRIC		E	MRI	1.5T	11	7M;4F	R	Talairach	spm99	MR self>control	11
				Motor/EGOCENTRIC		E	MRI	1.5T	11	7M;4F	R	Talairach	spm99	MR self>object	5
				Visual										MR object>self	8
57	Wraga et al., 2010a	3D cubes	“participants were asked to imagine Holding the object in their right (i.e., dominant) hand and mentally rotate the object”	MOTOR/Egocentric	M		MRI	3T	18	8M;10F	R	Talairach	spm2	MR obj in hand. fixation	7
			“Participants were asked to imagine rotating their bodies around the sphere until their eyes were aligned behind the horizontal line of the floating T-prompt and their noses were aligned behind the vertical line of the floating T-prompt”	MOTOR/Egocentric		E	MRI	3T	18	8M;10F	R	Talairach	spm2	MR body>fixation	6
58	Wraga et al., 2010b	3D cube plus cue	Body minimize: participants imagined rotating themselves to the location of the T promt floating outside the sphere and pressed yes no button to indicate whether the textured portion of the object was visible	MOTOR/Egocentric		E	MRI	3T	13	6F;7M	R	Talairach	spm2	Boby minimize -fixation	5
			Body maximize: participants performed the same imagined transformation, but pressed left or right buttons that served as virtual pointers to indicate whether the textured portion of the object was to their right or left	MOTOR/Egocentric		E								Body maximize- fixation	8
59	Zacks et al., 2002	Body pair	Same different task: two bodies were presented; varied which arm was extended (left or right): decide if the two figures were identical (same) or mirror images (different). As defined by instructions and by the authors; In the left–right task: participants were instructed to decide if the figure's left or right arm was extended	MOTOR/EGOCENTRIC		E	MRI	1.5T	18	5M;12F	R	Talairach	Not spec	MR>control	28
60	Zacks et al., 2003	Arrays of four blocks mounted on wooden posts at the corner of a square wooden board	An object was cued and the participant was asked to report the location of the object after an imagined viewer transformation	MOTOR/EGOCENTRIC		E	MRI	1.5T	16	2M;14F	R	Talairach	other	MR viewer>control	1
	Zacks et al., 2003		An object was cued and the participant was asked to report the location of the object after an imagined object transformation	VISUAL/ALLOCENTRIC										MR objects>control	1

MR can be accomplished taking as a reference frame the object itself (i.e., allocentric view) or the viewer's position (i.e., egocentric view). We considered also the reference frame effect, where it was possible, i.e., as indicated by the authors' instructions. For instance, all the studies in which a mental change of the whole body position in space (a self-rotation) is required (e.g., “[…] after having imagined themselves to be in the figure's body position”; “subjects updated the position of one of four external objects from memory after they had performed an imagined self-rotation to a new position”; see for instance in Wraga et al.'s (2005): “[…] imagined rotating themselves to the location of the T-prompt,” or in Creem-Regehr et al.'s (2007) “[…] instructed to imagine that they were standing at the blue sphere, and from that new imagined perspective to decide whether the previously named hand part, “thumb” or “pinky,” was on their right or left”) were included under the category “egocentric.” Activation in the first group (motor strategy) is expected to be left-lateralized as it exercises processes that prepare motor movements, and it might reflect the left hemisphere dominance for action and goal-directed motor behavior (and apraxia).

The activation in the brain while solving a MR task can be modulated by the type of stimulus. For instance, neuropsychological studies indicate that different operations may be recruited in MR depending on whether the stimulus type is a body part or a two or three-dimensional object ^2^. In particular it has been shown that lesions in the left hemisphere impaired MR of hands, while lesions in the right hemisphere affected MR of external objects (e.g., a puppet and flag shapes) (Tomasino et al., 2003b). For instance, some authors (Kosslyn et al., 1998) directly compared different types of stimuli and showed that MR of 3D cubes enhanced bilateral activation in the right parietal lobe and in BA 19, whereas MR of hands enhanced unilateral left activation in the precentral gyrus (M1), most of the parietal lobe, the primary visual cortex, the insula, and frontal premotor cortex (BA 6) and the superior frontal cortex (BA 9). The authors proposed that MR of hands and objects can be carried out by engaging two independent mechanisms: one requiring processes that prepare motor movements, and one that does not. Lastly, it has also been shown that performing MR by imagining rotating Shepard and Metzler's stimuli as a consequence of subjects' own hand action (i.e., motor strategy) elicited activation in the left primary motor cortex—the region that in Kosslyn's PET study (Kosslyn et al., 1998) was activated in association with MR of hands only as compared to performing MR by imagining what one would see if someone else, or an external force, manipulated an object (i.e., external strategy) because they simulated a manual rotation (see also Wolbers et al., 2003; Wraga et al., 2003). In a neuropsychological study it was shown that independent of the stimulus to be rotated, patients with right hemisphere lesions were found to be selectively impaired in performing MR by using a visual strategy but were still able to perform MR based on the motor strategy. By contrast, patients with left hemisphere lesions were found to be selectively impaired in MR based on the motor strategy, with intact visual strategy based MR (Tomasino and Rumiati, 2004).

In the present study, we performed quantitative activation-likelihood-estimation (ALE) meta-analyses (Turkeltaub et al., 2002; Laird et al., 2005, 2009; Eickhoff et al., 2009) of functional neuroimaging experiments on MR. We tested a previously formulated hypotheses, the top-down and bottom up hypothesis formulated in published works (Tomasino and Rumiati, 2004, 2013; Tomasino et al., 2004, 2011; Papeo et al., 2012). We referred to bottom-up factors as the effect exerted by the type of stimulus under rot ation. Presenting body parts or external objects as stimuli might differentially contribute to the MR network. In addition, we referred to top-down factors as the effect exerted by the type of MR strategy required by MR instructions. We first identified the MR network including areas consistently activated in neuroimaging studies addressing MR abilities. In a previously published quantitative meta-analysis on MR literature (Zacks, 2008) the network related to MR included the intraparietal sulcus bilaterally, the precentral sulcus bilaterally, the left occipital lobe, and the cingulate gyrus. That meta-analysis and the present study differ in the following aspects. First, in Zack's study, aside a modest number of studies included (data from 32 articles and 320 activation foci, vs. 60 articles and 884 activation foci in the present study), the research question addressed was whether MR depends on analog spatial representations or on motor simulation, whereas here we address the influence of the type of stimulus or reference frame on the MR network. Second, in this previous meta-analysis the author reports that the study search in literature has been done in Medline till 2006. As analysis method to generate the final maps he used volume-wise probability maps (the method and software described by Turkeltaub et al., 2002). We used here a more recently revised activation likelihood estimation (ALE) method, (Eickhoff et al., 2009). Third, the aim of Zack's study was to investigate whether MR depends on analog spatial representation, and whether MR depends on motor simulation. To do so the studies were divided in omnibus and in transformation specific type of MR. On the contrary, in the present study, to address how the MR network can be modulated by the type of stimulus and strategy we divided the studies in the following subgroups: bodily and non-bodily stimuli and motor imagery based vs. visuo-spatial (non-motor)-imagery based transformations. Lastly, Zacks reported the main MR activation network; in the present study we aimed at reporting: the main MR activation network (with a higher number of studies included see above), the MR of bodily stimuli network, the MR of non-bodily stimuli network; the MR motor strategy network; and the MR visual strategy network.

Using an MR task that, through instructions, requires using a motor-imagery based strategy or a visual-imagery based strategy, might differentially contribute to the MR network, as well as the type of stimulus (bodily- and non-bodily-related MR). Based on previous neuropsychological studies (Tomasino et al., 2003a,b), we expected to find that hands and body stimuli will preferentially elicit consistent fMRI activations in the sensorimotor network, whereas 3D cubes, objects, alphanumeric characters and abstract characters will preferentially activate a right-hemisphere network of areas since the right hemisphere is held to be involved in spatial operations (e.g., Ratcliff, 1979; Farah et al., 1988). As a further issue, we addressed whether MR of body-part and MR of whole body can be distinguished in terms of fMRI activation. Whole body pictures require an imagined transformation of one's own body, whereas MR of hands do not.

Testing how the type of stimulus, i.e., bottom up modulation, can modulate the MR-related activations can shed light on some conflicting imaging results showing that MR is lateralized to the left (Alivisatos and Petrides, 1997; Zacks et al., 1999; Kosslyn et al., 2001; Vingerhoets et al., 2001) or to the right hemisphere (Harris et al., 2000; Podzebenko et al., 2002, 2005) or bilaterally (Cohen et al., 1996; Richter et al., 1997; Tagaris et al., 1997; Kosslyn et al., 1998; Carpenter et al., 1999; Jordan et al., 2001).

Testing how the type of strategy, i.e., top down modulation, can modulate the MR-related activations can shed light on the debate about whether the sensorimotor cortex is involved in MR. Finally, some neuroimaging studies explained that activation found in M1 during MR was only due to the action of subjects responding by pressing the response button (Cohen et al., 1996; Richter et al., 2000), others claimed that it provides evidence for the involvement of M1 in MR (Tagaris et al., 1998; Kosslyn et al., 1998; Carpenter et al., 1999; Ganis et al., 2000; Lamm et al., 2001; Vingerhoets et al., 2001). Still others failed to report any M1 activation when subjects performed MR tasks (Parsons and Fox, 1998; Barnes et al., 2000; Harris et al., 2000; Jordan et al., 2001). The presence or absence of M1 activation may be dependent upon the type of stimuli and strategy used and the nature of the stimulus can have a role in triggering the motor or the visual strategy.

## Materials and methods

We filtered the PubMed database (www.pubmed.org), the Web of Knowledge database (www.webofknowledge.com), and the Sleuth on-line database (http://brainmap.org), for functional neuroimaging experiments that investigated MR processes. Moreover, the literature cited in the selected papers and reviews was also searched for additional neuroimaging studies on MR. The included studies were PET or fMRI experiments carried out on healthy subjects. Studies involving pharmacological trials or clinical populations were excluded. They were analyzed by means of a random-effects analysis. Analyses based on regions of interest (ROIs) of functional localizers were excluded. All single-cases studies were excluded, too. In addition, only studies which reported the coordinates in a standard reference space (Talairach/Tournoux, MNI) were considered. Differences in coordinate spaces (MNI vs. Talairach space) were accounted for by transforming coordinates reported in Talairach space into MNI coordinates using a linear transformation (Lancaster et al., 2007).

Based on these criteria, we selected 51 fMRI papers and 9 PET papers for a total of 171 included experiments in which the MR paradigm had been used. Table 1 provides a description of all the included studies. We divided all the collected experiments into several groups: stimuli and strategies/reference frame (Table 1). In the first classification, the criteria we used to classify “body” (i.e., hands or human body) vs. “non-body” (i.e., alphanumeric characters, 3D cubes, abstract stimuli) related stimuli was how the authors described the stimuli. In particular, in the body part category, hand shapes, full bodies, hands and arm pictures were labeled and classified as body part and included. Whereas in the non-bodily category we included all the other stimuli, namely alphanumeric characters, 3D cubes, abstract stimuli. In this classification graspable tools were not included in either body part group or non-bodily stimuli group as they can be grasped thus could be thought of as related to the body, but still they are not body parts. The criteria we used to classify “motor” vs. “visual” strategy was the task instructions reported by authors in the different studies (see Table 1, column 4). For instance Study n.3 reports that authors instructed participants to “visualize it rotating in a continuous movement until it aligned with the other figure.” This type of instruction corresponds to a visual strategy of visualizing the stimulus rotating. Studies in which no explicit instructions were used were excluded from the strategy-related analysis. In the second classification, we reported the specific instructions/definition of the paradigm used as defined by the authors of the included studies. In particular, studies for which detailed instructions or paradigm definition were given, were classified as egocentric/motor imagery based MR tasks and allocentric/visuo-spatial imagery based paradigms. MR of hands was labeled as egocentric/motor imagery based MR since in literature this type of MR is by definition solved via egocentric/motor imagery transformations.

Accordingly we included studies involving bodily-related stimuli (hands, bodies: 16 studies, 220 subjects) and non-bodily related stimuli (3D cubes, alphanumeric characters and abstract shapes: 55 studies, 722 subjects), motor imagery based MR (38 studies, 500 subjects) and visuo-spatial imagery based MR (22 studies, 264 subjects). Studies included for each of these categories are indicated in Table 1 (column Stimuli and column Strategy). We excluded studies in which no coordinates were reported (*N* = 62), pathological subjects were included (*N* = 39), pharmacological treatment was performed (*N* = 10), ROI analyses were carried out (*N* = 17), children (under 18) were included (*N* = 9), single cases were included (*N* = 5) and other (*N* = 9: 2 engaged transsexuals participants, 1 addressing comparison of women in mid-luteal phase and men, 1 addressing MR in experts in maths, 1 involving a acustic distractor task during MR, 1 including a ROI analysis, 1 engaging a task in which the stimulus disappeared and participants had to keep the mental image active in their mind before MR, 1 involving MR of tactile stimuli, and 1 in which participants mentally visualized the stimuli (they were not visually presented) through verbal instructions).

The reported coordinates for functional activation in each category were analyzed for topographic convergence using the ALE method.

## Statistical analysis

A meta-analysis was carried out using the revised version (Eickhoff et al., 2009) of the ALE approach for coordinate-based meta-analysis of neuroimaging results (Turkeltaub et al., 2002; Laird et al., 2005). To account for the uncertainty that is technically inherent to the actual location of the peaks, the method allows to model each coordinate not as a single point, but by a three-dimensional (3D) Gaussian function with 12 mm FWHM (Laird et al., 2005, 2009; Eickhoff et al., 2009). Accordingly, the localization probability distributions describe the probability that a given focus actually lay within a particular voxel (Laird et al., 2005, 2009; Eickhoff et al., 2009, 2012). ALE probability maps were then thresholded at *p* < 0.05 (cluster level corrected for multiple comparisons) (Laird et al., 2005, 2009; Eickhoff et al., 2009, 2012) and a minimum cluster size of 200 mm^3^ was set. We performed the following ALE analysis.

### ALE of all studies identifying the MR network

In the first ALE analysis we addressed the “MR Network” by including all eligible studies in order to assess the general MR brain network, by determining brain areas with consistent activation across all studies on MR considered.

### ALE of studies grouped by stimulus type [bodily > non-bodily related stimuli and reversed contrast)]

To explore how the MR network is influenced by the type of stimulus (see above), the reported studies were grouped as i) bodily- (i.e., hands and body) and non-bodily (i.e., 3D cubes, objects, alphanumeric characters and abstract characters) related stimuli. In addition, we included an analysis comparing MR of hands and MR of body stimuli. In Table 1, we indicated the studies with body or hand stimuli in a corresponding column.

### ALE of studies grouped by paradigm type [motor imagery-/egocentric > visuo-spatial imagery/allocentric and reversed contrast]

In a third analysis, we explored how the MR network is influenced by egocentric/motor-imagery based strategies and allocentric/visual-imagery based strategies. In addition, we included an analysis by distinguishing the strategy from the reference frame variable. We thus compared motor-imagery based MR, see in Table 1 the studies indicated with M in the column Motor only and egocentric MR, see in Table 1 the studies indicated with E in the column egocentric only). An example of motor strategy can be found in Corradi-Dell'Acqua et al.'s study (2009) in which authors report that during the MR task “participants were asked to accomplish the task using different strategies, that is, either by imagining the arm stimulus rotating until this could be wedged in the human photograph (visual strategy) or by ignoring the human photograph and imagining to rotate their own arm until this reached the position depicted in the screen (motor strategy).” An example of egocentric MR can be found in Creem et al.'s study (2001) in which subjects “updated the position of one of four external objects after they had performed an imagined self-rotation to a new position.”

### ALE of studies grouped by single stimulus vs. pair of stimuli presentation

Lastly, we included an additional analysis comparing MR of single stimulus or pairs of stimuli (see in Table 1 the studies indicated with pair in the column Stimuli)^3^.

Activations were assigned using the SPM Anatomy Toolbox (Eickhoff et al., 2005).

## Results

### MR network

**Figure 1:**
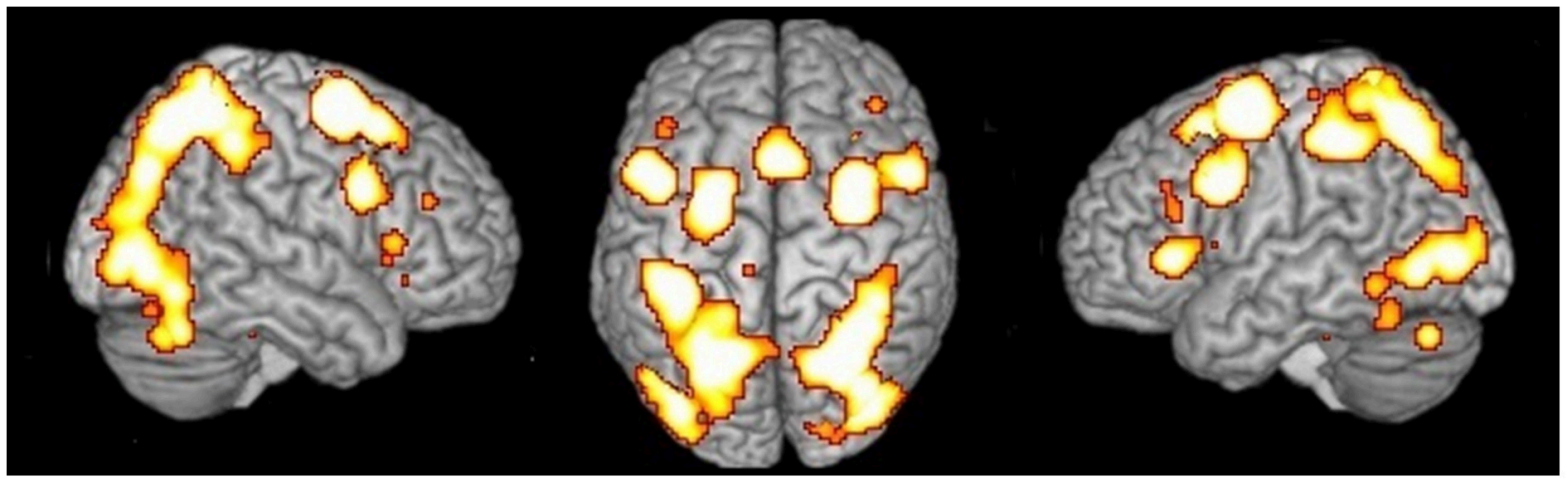
**Network of activations underlying MR**. Relative increases in neural activity associated with MR are displayed on a rendered template brain provided by spm5. Activations are significant at *p* < 0.05 corrected for multiple comparisons using the False Discovery Rate (FDR).

Independent of the type of stimulus or the type of strategy, the MR network included activations in the: (i) inferior and superior parietal lobule bilaterally; (ii) left precentral gyrus; (iii) inferior frontal gyrus bilaterally; (iii) middle frontal gyrus bilaterally; (iv) SMA; (v) left insula; (vi) inferior and middle occipital gyrus bilaterally, and (vii) cerebellum bilaterally (see Table 2 and Figure 1).

**Table 2:** **Results of the ALE meta-analysis revealing the MR network**.

**Cluster**	**Area**	**MNI coordinates**			**Cluster size (voxels)**	**Extreme value**
		***X***	***Y***	***Z***		
**MR NETWORK**						
1	L Inferior Parietal Lobule (Area 2)	−40	−38	46	2954	0.067
	L Superior Parietal Lobule (Area7A)	−18	−64	52		0.061
2	R Superior Parietal Lobule (Area 7A)	28	−62	52	2877	0.071
	R Inferior Parietal Lobule (hIP3)	38	−42	44		0.041
3	L Middle Frontal Gyrus (Area 6)	−26	−4	56	1679	0.072
	L Inferior Frontal Gyrus (p. Opercularis) (Area 44)	−46	6	28		0.045
	L Precentral Gyrus (Area 6)	−40	−4	48		0.017
4	R Middle Frontal Gyrus (Area 6)	30	−4	56	897	0.089
5	L Inferior Occipital Gyrus (Area V5)	−48	−70	−6	739	0.040
	L Middle Occipital Gyrus (Area V5)	−38	−82	0		0.037
6	R Inferior Occipital Gyrus	44	−64	−16	605	0.033
	R Cerebellum	42	−62	−30		0.030
7	R Inferior Frontal Gyrus (Area 44)	52	10	24	559	0.040
8	R SMA (Area 6)	4	14	48	547	0.048
9	R Inferior Occipital Gyrus (V3v)	32	−86	−6	441	0.030
	R Middle Occipital Gyrus (V5)	42	−78	4		0.024
10	L Insula	−32	26	−2	287	0.038
11	L Middle Frontal Gyrus (Area 45)	−44	32	28	102	0.020
	L Inferior Frontal Gyrus (p. Triangularis) (Area 45)	−44	26	16		0.018
12	R Middle Frontal Gyrus	40	36	22	100	0.027
13	L Cerebellum	−42	−74	−32	67	0.029
14	L Cerebellum	−36	−58	−24	63	0.020
15	L Fusiform Gyrus	−30	−54	−12	44	0.021
16	R Lingual Gyrus (Area 17)	18	−90	−4	37	0.019

*Peaks of activation corrected above the threshold, MNI Coordinates (x, y, z) of maximum ALE-value, and maximum ALE-value of this cluster. All peaks are assigned to the most probable brain areas as revealed by the SPM Anatomy Toolbox (Eickhoff et al., 2005)*.

### Type of stimulus dependent modulation: ALE of studies grouped by stimulus type

A bilateral sensorimotor activation and a right lateralized activation were found for bodily- and non-bodily stimulus respectively.

#### MR of bodily > MR of non-bodily related stimuli (and viceversa)

**Figure 2:**
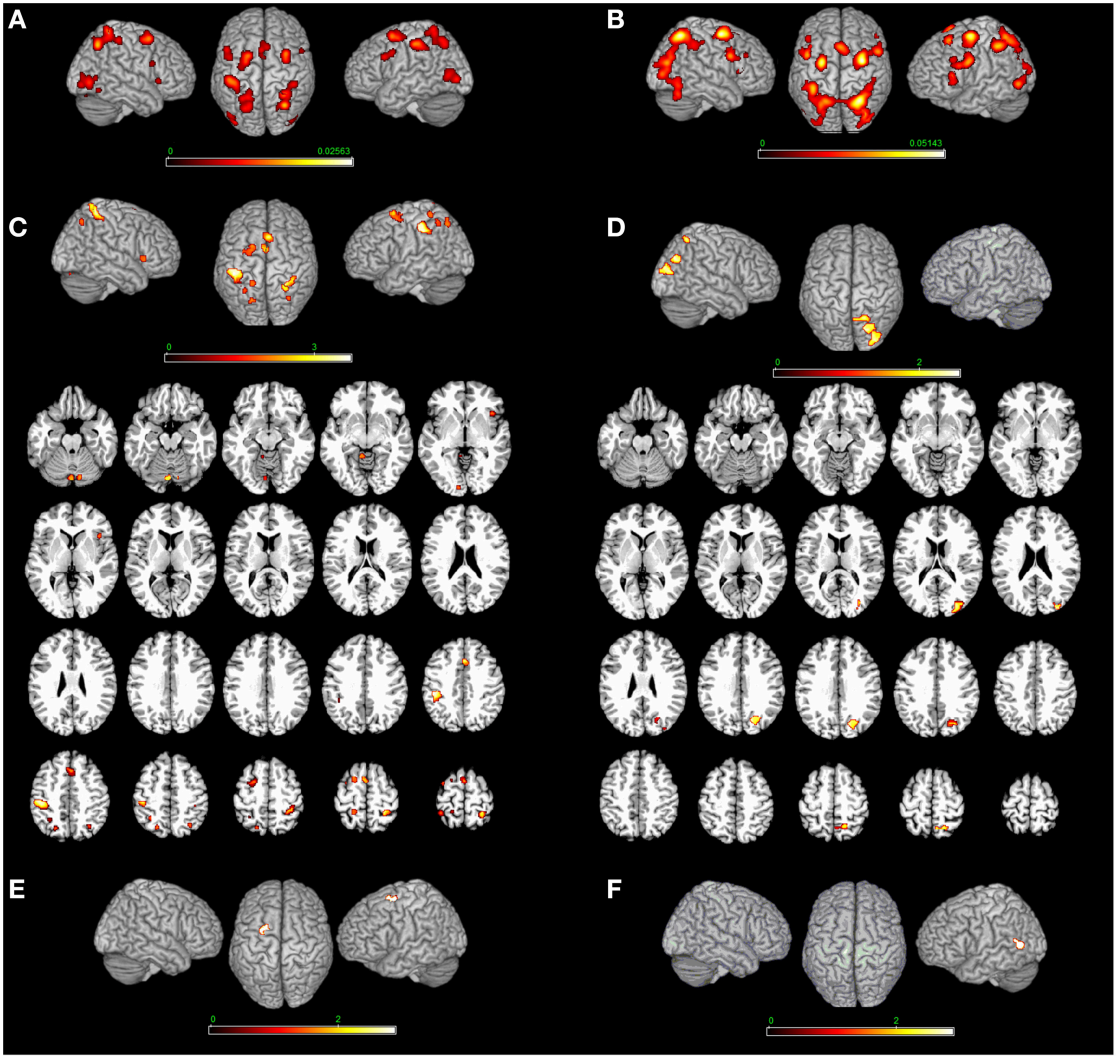
**Bottom-up modulation of the MR network exerted by the type of stimulus**. Bodily- **(A)** and non-bodily **(B)** stimulus. **(C)** Shows the direct contrast bodily> non-bodily stimulus and **(D)** shows the contrast non-bodily—bodily stimulus. For bodily related stimuli, in **(E)** we report MR of hands > body and in **(F)** we report MR of body—hands. Relative increases in neural activity associated with MR induced by different types of stimuli are displayed on a rendered template brain provided by spm5. Activations are significant at *p* < 0.05 corrected for multiple comparisons using the False Discovery Rate (FDR). In **(A,B)** color bar shows ALE value, in **(C–F)** color bar shows Z maps. The Z coordinates for each slices range from −24 to 66 (with incremental steps of 5 mm).

MR of bodily as compared to non-bodily related stimuli included activations in the: (i) cerebellum bilaterally and left calcarine cortex, (ii) left inferior parietal lobe, right angular gyrus, left superior parietal lobe and right postcentral gyrus (Areas 2, 3b, and 4p), (iii) right insula, left superior frontal gyrus and middle cingulate cortex. The reverse contrast (non-bodily related > bodily-related stimuli) included exclusively right-lateralized activations in the: (i) middle occipital gyrus, (ii) cuneus, and (iii) superior parietal cortex (see Table 3 and Figure 2).

**Table 3:** **Results of the ALE meta-analysis from the direct contrasts revealing the bottom-up modulation of the MR network exerted by the type of stimulus and strategy**.

**Cluster**	**Area**	**MNI coordinates**			**Cluster size (voxels)**
		***X***	***Y***	***Z***	
**MR OF BODILY- NON-BODILY STIMULI**					
1	L Inferior parietal lobe	−45.2	−31.6	40	291
2	R Postcentral gyrus (Areas 2, 3b, 4p)	26.35	−48.19	66.1	194
3	L Superior frontal gyrus	−20	8	62	107
4	M Middle cingulate cortex	3	19	40	97
5	R Cerebellum	0	−80	−20	72
6	L Posterior medial frontal gyrus	−6	4	63	62
7	L Superior parietal lobe	−20	−68	46	46
8	R Insula	40	22	−4	45
9	L Superior parietal lobe	−18	−46	64	39
10	R Cerebellum	12	−80	−20	36
11	L Cerebellum	−10	−46	−14	34
12	R Angular gyrus	30	−66	48	28
13	L Superior parietal lobe	−34	−50	68	28
14	L Calcarine cortex	−10	−93	−8	26
15	L Inferior parietal lobe	−30	−54	52	26
**MR OF NON-BODILY—MR OF BODILY STIMULI**					
1	R Middle occipital gyrus	32	−90	20	173
2	R Cuneus	18	−76	32	145
3	R Superior parietal lobule, precuneus	16	−60	58	83
**MOTOR-IMAGERY BASED/EGOCENTRIC MR—VISUO-SPATIAL IMAGERY BASED/ALLOCENTRIC MR**					
1	R Postcentral gyrus (Areas 2, 3b, 4p)	24	−44	62	215
2	L Inferior parietal lobe, postcentral gyrus (Areas 2, 1 3b)	−44	−30	40	177
3	L Superior parietal lobe	−18	−50	70	92
4	R Angular gyrus	34	−64	48	43
**VISUO-SPATIAL IMAGERY BASED/ALLOCENTRIC MR—MOTOR-IMAGERY BASED/EGOCENTRIC MR**					
1	R Precuneus	16	−54	48	186
2	R Superior frontal gyrus	22	−12	52	124
3	R Superior occipital gyrus	28	−70	28	91
4	L Middle occipital gyrus	−32	−89	13	76
5	L Superior parietal lobe	−38	−64	58	48
6	L Inferior temporal gyrus, inferior occipital gyrus	−50	−68	−10	46
7	L Middle occipital gyrus	−28	−72	32	27
8	R Posterior medial frontal gyrus	8	10	54	26
**MR OF HANDS—MR OF BODY**					
1	L Precentral gyrus (Area 6)	−20	−2	59	180
**MR OF BODY—MR OF HANDS**					
1	L Lingual gyrus (Area 18)	−15	−69	−1	77
**MOTOR-IMAGERY BASED-EGOCENTRIC MR**					
1	R Middle frontal gyrus/precentral gyrus (Area 6)	29	12	52	449
2	L Superior parietal lobule	−22	−56	66	343
3	L Superior frontal gyrus/precentral gyrus (Area 6)	−22.8	−4.8	58.4	273
4	L Superior parietal lobule	−25	−72	41	125
5	R Postcentral gyrus (areas 1, 2, 3b)	34	−42	66	107
6	L Inferior occipital gyrus	−42	−70	−4	98
**EGOCENTRIC MR- MOTOR-IMAGERY BASED**					
1	L Cuneus	−12	−79	20	109
2	L Middle temporal gyrus	−61.91	−52.15	−1.12	68
3	L Calcarine gyrus, Linual gyrus	−15	−65	5	36
4	L Cuneus	−2	−80	22	34
5	R Cerebellar vermis	4	−73	−26	31
**PAIR OF STIMULI-SINGLE STIMULI**					
1	L IFG (p. Opercularis)	−46.67	9.33	20.67	216
2	L Middle frontal gyrus	−25.67	8.33	63	209
3	R Middle occipital gyrus	42	−83	0	345
4	L Superior parietal lobule	−20	−47.6	65.2	124
5	L Middle occipital gyrus	−30	−70	34	46
6	L IFG (p. Triangularis)	−43	27	13	37
7	L Cerebelum	−38	−74	−26	34
**SINGLE STIMULI—PAIR OF STIMULI**					
1	R Inferior/Superior parietal lobule	32	−48	48	238
2	R Cerebellar vermis	6	−73	−16	111
3	L Middle occipital gyrus	−38	−86	26	79
4	L Middle occipital gyrus	−31	−95	10	63
5	R IFG (p. Opercularis)	38	8	34	63
6	L Middle frontal gyrus	−18	−6	48	50
7	L Inferior parietal lobule	−40	−42	38	45
8	R Precuneus	12	−70	50	40
9	L Precuneus	−12	−56	52	39
10	L (L Cerebelum (Crus 1)	−40	−50	−30	36
11	R Middle frontal gyrus	28	4	44	31
12	L Superior medial gyrus	−4	18	42	29
13	L Paracentral lobule 4a	−8	−30	66	25

*Peaks of activation corrected above the threshold, MNI Coordinates (x, y, z) of maximum ALE-value, and maximum ALE-value of this cluster. All peaks are assigned to the most probable brain areas as revealed by the SPM Anatomy Toolbox (Eickhoff et al., 2005)*.

#### MR of bodily stimuli

MR of bodily stimuli included activations bilaterally in the: (i) cerebellum, middle and inferior occipital and calcarine gyrus, (ii) superior parietal lobule, postcentral gyrus (Area 2) bilaterally, left postcentral gyrus (Area 1), left inferior parietal lobe and right supramarginal gyrus, (iii) left precentral gyrus and inferior frontal gyrus (pars opercularis) bilaterally, left superior frontal gyrus, right middle frontal gyrus and posterior frontal gyrus medially, in addition to the right insula (see Table 4 and Figure 2). Regarding the comparison between hand and body stimuli, results showed that MR of hands (vs. MR of body stimuli) activated the left precentral gyrus (Area 6). MR of body stimuli (vs. MR of hands) activated the left lingual gyrus (Area 18) (see Table 3 and Figure 2).

**Table 4:** **Results of the ALE meta-analysis revealing the main effect of the type of stimulus and strategy**.

**Cluster**	**Area**	**MNI coordinates**			**Cluster size (voxels)**	**Extreme value**
		***X***	***Y***	***Z***		
**MR OF BODILY STIMULI**						
1	R Superior parietal lobe, postcentral gyrus (Area 2)	28	−64	50	513	0.0228
2	L Superior frontal gyrus	−26	−6	54	429	0.0213
3	L Postcentral gyrus (Area 2)	−40	−34	46	424	0.0255
4	L Superior parietal lobe, inferior parietal lobe	−20	−66	52	377	0.0181
5	R Middle frontal gyrus	30	0	54	312	0.0222
6	M Posterior medial frontal gyrus	−4	12	48	240	0.0174
7	L Precentral gyrus, inferior frontal gyrus (pars opercularis)	−48	2	32	199	0.0161
8	L Cerebellum	−4	−80	−20	76	0.0124
9	M Posterior medial frontal gyrus	−2	2	62	65	0.0128
10	R Middle occipital gyrus, inferior occipital gyrus	38	−86	2	59	0.0119
11	R Inferior frontal gyrus (pars opercularis)	52	10	20	54	0.0126
12	R Inferior occipital gyrus	42	−64	−16	49	0.0138
13	R Insula	42	20	−2	45	0.0140
14	L Superior parietal lobe (Areas 2, 1)	−20	−46	60	39	0.0127
15	R Cerebellum	12	−78	−24	37	0.0119
16	L Cerebellum	−8	−46	−10	34	0.0109
17	R Calcarine gyrus	8	−76	10	31	0.0115
18	R Middle frontal gyrus	38	36	20	31	0.0125
19	L Supramarginal gyrus	−58	−24	20	28	0.0112
20	L Superior parietal lobe (Area 1)	−36	−48	68	28	0.0126
21	L Calcarine gyrus	−10	−94	−4	27	0.0120
22	L Middle occipital gyrus	−38	−88	−2	27	0.0112
**MR OF NON-BODILY STIMULI**						
1	R Superior parietal lobe, inferior parietal lobe, middle occipital gyrus	26	−60	54	4863	0.0491
2	R Middle frontal gyrus	30	−4	56	712	0.0512
3	L Superior frontal gyrus	−26	−6	60	604	0.0448
4	L Inferior frontal gyrus (pars opercularis and triangularis)	−46	4	28	511	0.0320
5	L Inferior temporal gyrus, inferior occipital gyrus, middle occipital gyrus	−50	−68	−8	476	0.0295
6	M Posterior medial frontal gyrus	0	14	50	347	0.0305
7	R Inferior frontal gyrus (pars opercularis) Precentral	52	8	26	359	0.0305
8	L Insula	−30	22	6	189	0.0242
9	R Insula	30	22	4	68	0.0196
10	L Middle frontal gyrus	−44	26	32	55	0.0176
11	R Precentral gyrus	42	6	34	30	0.0159
12	R Inferior frontal gyrus (pars triangularis)	50	30	26	30	0.0161
13	L Cerebellum	−36	−60	−24	27	0.0155
**MOTOR STRATEGY**						
1	R Angular gyrus, superior parietal lobe, inferior parietal lobe, postcentral gyrus (Area 2)	28	−64	48	1103	0.0386
2	L Superior parietal lobe	−22	−54	66	832	0.0254
3	L Superior frontal gyrus, precentral gyrus	−22	4	56	610	0.0358
4	L Postcentral gyrus (area 2)	−46	−32	46	436	0.0287
5	R Inferior occipital gyrus, middle occipital gyrus	42	−76	−6	378	0.0223
6	L Middle occipital gyrus	−38	−88	−4	360	0.0223
7	R Middle frontal gyrus	30	6	56	356	0.0317
8	L Inferior frontal gyrus (pars opercularis), precentral gyrus	−50	12	26	337	0.0262
9	M Posterior medial frontal gyrus	4	14	44	158	0.0181
10	R Cerebellum	8	−74	−22	129	0.0172
11	R Middle occipital gyrus	32	−74	34	83	0.0169
12	M Posterior medial frontal gyrus	−2	4	62	78	0.0188
13	R Inferior frontal gyrus (pars opercularis)	52	12	20	44	0.0153
14	L Middle frontal gyrus	−28	44	18	28	0.0134
15	R Insula	42	20	−4	25	0.0145
**VISUO-SPATIAL STRATEGY**						
1	R Superior parietal lobe, middle occipital gyrus, inferior parietal lobe	24	−62	52	981	24
2	L Superior parietal lobe, inferior parietal lobe, precentral gyrus, middle occipital gyrus	−20	−62	54	629	−20
3	R Precentral gyrus, middle frontal gyrus, superior frontal gyrus	30	−6	56	549	30
4	L Superior frontal gyrus	−26	−8	60	480	−26
5	M Posterior medial frontal gyrus	−4	16	48	232	−4
6	L Inferior temporal gyrus	−50	−68	−6	147	−50
7	L Inferior parietal lobe	−38	−48	46	117	−38
8	R Precentral gyrus	56	12	36	82	56
9	L Middle occipital gyrus	−28	−86	12	80	−28
10	L Cerebellum	−42	−74	−32	63	−42
11	R Inferior occipital gyrus	42	−76	−10	59	42
12	R Postcentral gyrus (Area 2)	46	−28	44	53	46
13	R Inferior frontal gyrus (pars opercularis)	54	14	22	46	54
14	R Middle occipital gyrus	34	−90	16	32	34
15	R Cerebellum	42	−62	−32	30	42
16	L Insula	−30	22	10	27	−30

*Peaks of activation corrected above the threshold, MNI Coordinates (x, y, z) of maximum ALE-value, and maximum ALE-value of this cluster. All peaks are assigned to the most probable brain areas as revealed by the SPM Anatomy Toolbox (Eickhoff et al., 2005)*.

#### MR of non-bodily stimuli

MR of non-bodily related stimuli included activations in the: (i) middle occipital gyrus bilaterally, left inferior occipital gyrus, left cerebellum and left inferior temporal gyrus, (ii) right superior and inferior parietal lobe, and inferior frontal gyrus (pars opercularis and triangularis) bilaterally, in insula and the middle frontal gyrus in addition to the right precentral gyrus and the posterior frontal gyrus medially (see Table 4 and Figure 2).

### Type of strategy dependent modulation: ALE of studies grouped by paradigm type

A top-down modulation of the network was exerted by the MR strategy/reference frame with a preferentially sensorimotor network for motor imagery- vs. non-motor imagery-based MR and the latter activating a preferentially posterior occipito-temporal-parietal network as follows.

#### Motor > visual strategy (and viceversa)

**Figure 3:**
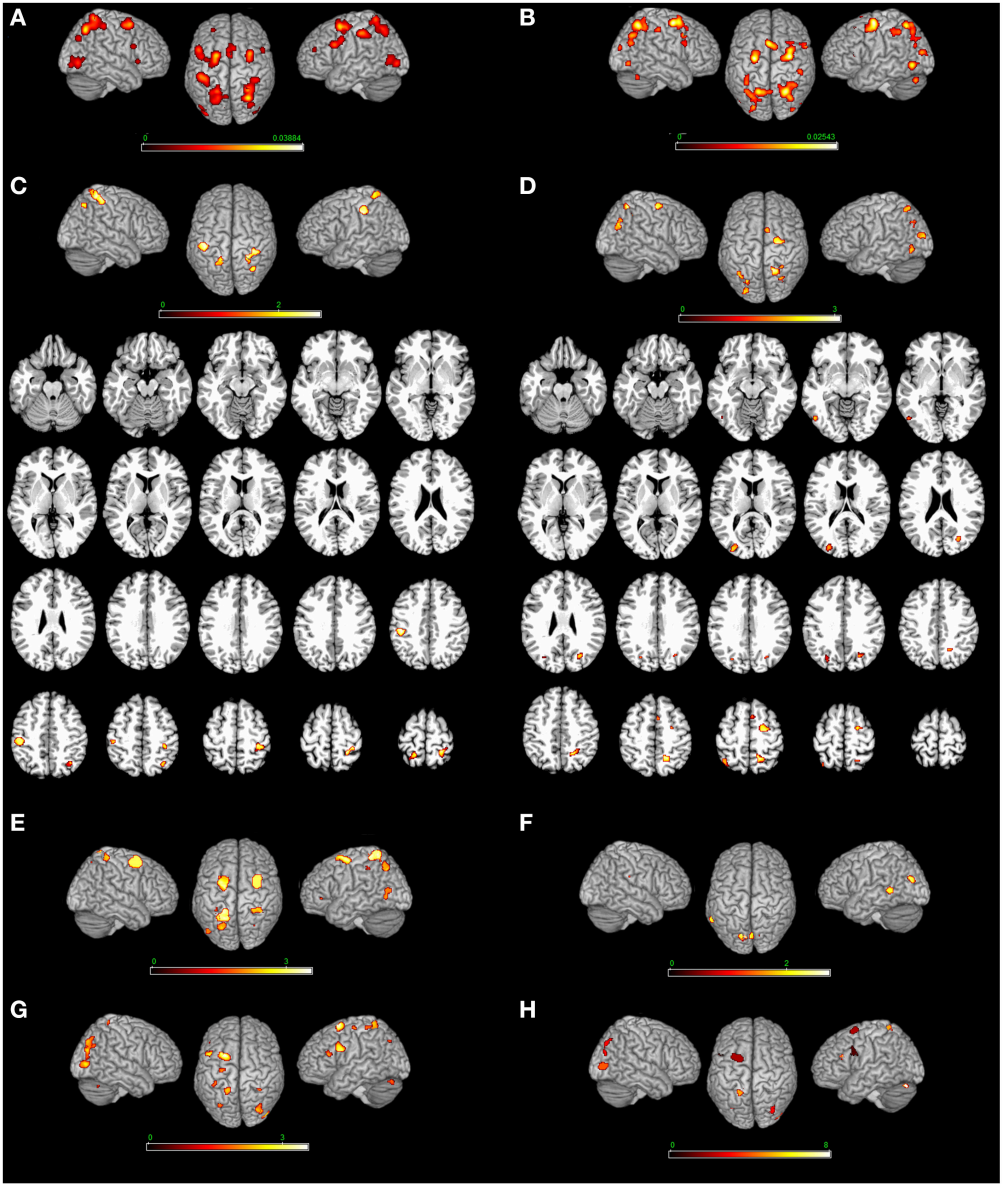
**Top-down modulation of the MR network exerted by the type of strategy**. [Motor imagery-based/egocentric **(A)** non-motor-imagery-based/allocentric **(B)** MR] and by the direct contrast [**(C)** motor imagery-based/egocentric > non-motor-imagery-based/allocentric and **(D)** non-motor-imagery-based/allocentric > motor imagery-based/egocentric]. **(E)** Motor-imagery based MR (vs. egocentric MR); **(F)** egocentric MR (vs. motor-imagery based MR); **(G)** Single stimulus (vs. pair of stimuli) presentation; **(H)** Pair of stimuli (vs. single stimulus). Relative increases in neural activity associated with MR induced by different types of strategies are displayed on a rendered template brain provided by spm5. Activations are significant at *p* < 0.05 corrected for multiple comparisons using the False Discovery Rate (FDR). In **(A,B)** color bar shows ALE value; in **(C–F)** color bar shows Z maps. The Z coordinates for each slices range from −24 to 66 (with incremental steps of 5 mm).

Motor strategy as compared to visual strategy included activations in the: (i) right postcentral gyrus (Areas 4p, 2, 3b), (ii) left postcentral gyrus (Areas 2, 1, and 3b) extending to the inferior parietal lobe, (iii) left superior parietal lobe and iv) the right angular gyrus. The reverse contrast (visual strategy as compared to motor strategy) included activations in the: (i) left middle occipital gyrus, left inferior occipital gyrus, left inferior temporal gyrus, and right superior occipital gyrus, (ii) right precuneus, (iii) left superior parietal lobe, and (iv) right posterior medial frontal gyrus and right superior frontal gyrus (see Table 3 and Figure 3).

#### Motor strategy

MR performed via the motor imagery based/Egocentric strategy included bilateral activations in the: (i) cerebellum bilaterally, (ii) right inferior occipital gyrus and middle occipital gyrus bilaterally, (iii) superior parietal lobe and postcentral gyrus (Area 2) bilaterally, (iv) left precentral gyrus, (v) inferior frontal gyrus (pars opercularis) bilaterally, (vi) left superior frontal gyrus and middle frontal gyrus bilaterally, (vii) posterior medial frontal gyrus, and (viii) right insula (see Table 4 and Figure 3). In addition, we performed an analysis by distinguishing the strategy from the reference frame variable. Motor-imagery based MR and egocentric MR, and we directly compared them. Results showed that motor-imagery based MR (vs. egocentric MR) activated the left superior parietal lobule, the right postcentral gyrus (Areas 1, 2, and 3b) and the precentral gyrus/middle and superior frontal gyrus bilaterally, and the left inferior occipital gyrus. Egocentric MR (vs. motor-imagery based MR) activated the left cuneus, the left middle temporal gyrus, the left lingual gyrus and calcarine sulcus, and the right cerebellum (see Table 3 and Figure 2).

#### Visual strategy

MR performed via the visuo-spatial imagery based/allocentric strategy included activations in the: (i) cerebellum bilaterally, (ii) middle occipital gyrus bilaterally, right inferior occipital gyrus and left inferior temporal gyrus, (iii) superior and inferior parietal lobe, (iv) left inferior parietal lobe, (v) right postcentral gyrus (Area 2), (vi) precentral gyrus bilaterally, (vii) superior frontal gyrus bilaterally, (viii) posterior medial frontal gyrus, (ix) right inferior frontal gyrus (pars opercularis), and (x) left insula (see Table 4 and Figure 3).

#### Single stimulus (vs. pair of stimuli) presentation

MR of pairs of stimuli (vs. single stimulus) included activations in the: (i) left inferior and in the middle frontal gyrus, (ii) left superior parietal lobule, and (iii) middle occipital gyrus (bilaterally) and left cerebellum. MR of single stimulus (vs. pair of stimuli) included activations in the: (i) right inferior frontal gyrus and in the middle frontal gyrus (bilaterally), (ii) right and left inferior, in the right superior parietal lobule, and precuneus bilaterally, (iii) left paracentral lobule (area 4a, at *x* = −8, *y* = −30, *z* = 66 approximately the foot area^4^), and (iv) left middle occipital gyrus and cerebellum bilaterally (see Table 3 and Figure 3).

## Discussion

Before addressing the implications of our main finding, that is, the differential modulation of the MR network exerted by the type of stimulus and by the type of strategy, we first discuss the neural network involved in the MR task *per se*. The activations encompassed areas which have been shown to be involved in MR processing by MEG, EEG, TMS, connectivity studies and neuropsychology (Kawamichi et al., 1998, 2007b; Tomasino et al., 2003a,b; Tomasino and Rumiati, 2004; Koshino et al., 2005; Feredoes and Sachdev, 2006; Mourao-Miranda et al., 2009; Seurinck et al., 2011; Lebon et al., 2012; Sack and Schuhmann, 2012; Thomas et al., 2013; Osuagwu and Vuckovic, 2014): the inferior and superior parietal lobule bilaterally, the precentral gyrus, the inferior frontal gyrus, the middle frontal gyrus, the SMA, the insula, the inferior and middle occipital gyrus and the cerebellum.

### Bottom-up modulation of the MR network by the type of stimulus

We found how the MR network can be modulated by the type of stimuli under rotation. A bilateral sensorimotor activation was found by comparing the bodily- to non-bodily stimuli. The network included the left inferior parietal lobe, right angular gyrus, left superior parietal lobe and right postcentral gyrus (Areas 2, 3b, and 4p), in addition to the left superior frontal gyrus and middle cingulate cortex. These findings are consistent with the TMS and neuropsychological literature on MR of hand shapes which seem to be related to the parietal lobe (Tomasino et al., 2003a,b; Pelgrims et al., 2005, 2009; Schwabe et al., 2009; Lebon et al., 2012; Yan et al., 2012, 2013; Thomas et al., 2013). In addition, we performed an analysis now by distinguishing MR of hands and MR of body stimuli, and we directly compared them. Results showed that MR of hands (vs. MR of body stimuli) activated the left precentral gyrus (Area 6). MR of body stimuli (vs. MR of hands) activated the left lingual gyrus (Area 18). These results confirm the view that whole body pictures and MR of hands can recruit different areas.

The reverse contrast (non-bodily related > bodily-related stimuli) included exclusively right-lateralized activations in the middle occipital gyrus, cuneus and superior parietal cortex. The literature on MR has shown that the key areas supporting MR of 3D cubes seem to be the superior parietal lobule (BA 7), together with the inferior frontal gyrus (BA 44/45) (Cohen et al., 1996; Thomsen et al., 2000), the middle frontal gyrus (BA 8), the parieto-occipital border (BAs 39/19) (Cohen et al., 1996) or –as in Richter et al. (2000)– the lateral premotor cortex (BA 6) and the supplementary motor area (medial BA 6). There are also studies showing a more intense activation in the right hemisphere (Richter et al., 1997; Thomsen et al., 2000) and others reporting a bilateral activation (Cohen et al., 1996; Tagaris et al., 1996, 1998; Carpenter et al., 1999; Richter et al., 2000). Similarly, the literature on MR of alphanumeric stimuli preferentially activated a right-lateralized network of areas involving the inferior and superior parietal lobule, the inferior temporal gyrus, the middle and inferior frontal gyrus, and the inferior and middle occipital gyrus, and MR of abstract stimuli included preferentially right-lateralized activations in the middle frontal gyrus and superior medial gyrus, the precuneus, the inferior and superior parietal lobule and the cerebellum. One cluster was found in the left hemisphere, in the left superior parietal lobule. These results are consistent with previous studies which showed that alphanumeric characters and abstract shapes enhance activation in the right superior parietal lobe (Alivisatos and Petrides, 1997; Tagaris et al., 1998; Harris et al., 2000; Podzebenko et al., 2002) together with the precentral gyrus more intensely over the right hemisphere, the extrastriate visual cortex (Tagaris et al., 1998), the left occipito-temporal junction (Podzebenko et al., 2002), the superior lateral cerebellum, the inferior frontal gyrus (BA 44/45) (Podzebenko et al., 2002) and the right posterior MFG (premotor area, BA 6) (Podzebenko et al., 2002).

Taken together, these results indicate that the type of stimulus used in MR experiments can elicit different patterns of activation and likely two types of MR mechanisms (Kosslyn et al., 1998).

A different account holds that it is the type of judgment that leads to the use of a given strategy. Tasks requiring participants to compare two simultaneously presented rotated images, in order to decide whether they are same or different, are likely to trigger object-based transformations. Whereas tasks requiring participants to judge whether a single stimulus, e.g., a body, shows the left or right arm raised are likely to trigger egocentric-based transformations (Steggemann et al., 2011). The possibility to classify studies according to this dichotomy was addressed by comparing MR of single stimulus or pairs of stimuli presentation, although the two variables, namely number of stimuli and type of transformation cannot be fully disentangled. One possibility could be comparing the ALE maps in the egocentric and one stimulus condition respectively the object-based and two stimuli condition. To perform this analysis a higher number of studies is necessary.

### Top-down modulation of the MR network by the type of strategy

The direct comparison between motor and visual strategy revealed the areas selectively modulated by one strategy or the other. In particular, when we directly compared the motor strategy to the visual strategy, we found bilateral activations in the sensorimotor areas. The reverse contrast (visual strategy as compared to the motor strategy) included bilateral activations involving the posterior occipital-temporal-parietal cortex. In addition, we performed an analysis by distinguishing the strategy from the reference frame variable. Motor-imagery based MR (vs. egocentric MR) activated the left superior parietal lobule, the right postcentral gyrus (Areas 1, 2, and 3b) and the precentral gyrus/middle and superior frontal gyrus bilaterally, and the left inferior occipital gyrus. Egocentric MR (vs. motor-imagery based MR) activated the left cuneus, the left middle temporal gyrus, the left lingual gyrus and calcarine sulcus, and the right erebellum. In a PET study (Kosslyn et al., 2001), subjects performed MR of Shepard and Metzler stimuli by imagining grasping the object and turning it with their own hand, and by mentally rotating the stimulus as if it were being rotated by a motor. The authors found an area of activation in the left superior/inferior parietal cortex, the left M1 cortex and the right parahippocampal gyrus when subjects solved the MR as a consequence of their manual activity (i.e., motor strategy). By contrast, the visual strategy activated the left inferior frontal gyrus (Area 47). The use of the motor and visual strategy in MR of 3D cubes has been further investigated in an fMRI study (Wolbers et al., 2003) by combining MR of 3D cubes and motor imagery for hands. The authors, who named the two strategies as active and passive rotations, detected an activation centered on the superior parietal lobe that was contralateral to the imagined hand.

Taken together, these results indicate that when requested by the experimenter through instructions subjects adopt one strategy or the other, and this triggers different modulation in the MR network in a top-down way.

One might argue that distinguishing visual and motor strategy is difficult even if a strategy is assigned by the authors of the original studies by means of instructions, since one cannot be sure of what the subjects do. However, in mostly all of the studies we included, authors reported behavioral data indicating that participants correctly used the motor or the visual strategy. For instance, in Papeo et al. (2012)'s study, it is reported that authors checked reliable indication that individuals used the motor or the visual strategy in RTs.

It is known that also the reference frame (egocentric or allocentric) modulates the MR processing. When directly comparing the strategy account to the reference frame variable we found that motor-imagery based MR (vs. egocentric MR) activated the left superior parietal lobule, the right postcentral gyrus (Areas 1, 2, and 3b) and the precentral gyrus/middle and superior frontal gyrus bilaterally, and the left inferior occipital gyrus, whereas the egocentric MR (vs. motor-imagery based MR) activated the left cuneus, the left middle temporal gyrus, the left lingual gyrus and calcarine sulcus, and the right cerebellum. These results indicate that these two mechanisms are different. Motor strategies engage motor behavior covertly whereas the egocentric based MR involves a (mostly spatial) judgment from another point of view (different from the perspective of the physical body).

The presentation of single stimulus vs. pairs of stimuli also influenced the MR network, however given the strength of the modulation exerted by the type of stimulus and the type of strategy we believe that it would be a very limited account considering the number of stimuli shown alone.

Lastly, the result related to the type of stimuli reporting a *bilateral* sensorimotor activation for bodily-related (vs. non-bodily-related stimuli) and a *posterior right* lateralized activation for non-bodily-related (vs. bodily-related stimuli) is consistent, as far as lateralization effects is concerned, with the result related to the type of strategy reporting a *bilateral* (preferentially sensorimotor left) network for motor imagery- vs. non-motor imagery-based MR and the latter activating a preferentially posterior *right* occipito-temporal-parietal network.

### Is the M1 cortex involved in MR?

Activation in M1 during MR tasks is not universally accepted. In some imaging studies the activation found in M1 during MR was explained as due to the subjects responding by pressing the response button (Cohen et al., 1996; Richter et al., 2000). Other studies have failed to report M1 activation when subjects performed MR tasks (Parsons et al., 1995; Parsons and Fox, 1998; Harris et al., 2000; Jordan et al., 2001), while others found M1 activated (Kosslyn et al., 1998). Further evidence supporting a critical role of the left M1 in MR is provided by Ganis et al. (2000) who, using TMS, showed that stimulating the human hand area in the left M1 at 650 ms after stimulus onset significantly slowed down the subjects' latencies when they mentally rotated hands, but not feet (Ganis et al., 2000). The idea that the manipulation of mental images is associated with a motor process was already intrinsic to the definition of MR given by Shepard and Cooper (1982). They pointed out that stimuli under MR appear to move in imagery, as they would if they were *physically rotated* by the subject. This operation can be triggered implicitly by hands, as confirmed by our meta-analysis. Indeed, in our meta-analysis M1 was found to be activated in the MR of bodily vs. non-bodily stimuli contrast (cluster 2) and it was found to be sensitive to the cognitive strategy used since it was again found in the motor-imagery/egocentric based strategy vs. visuo-spatial imagery/allocentric based strategy (cluster 1). We suggest that the type of stimulus (i.e., hands or external objects) may implicitly trigger one strategy or the other (i.e., motor or visual strategy, respectively), and that the left M1 supports the former. Results of the present meta-analysis are also consistent with neuropsychological data showing that MR can be impaired in patients with a tumor affecting the hand area, providing that they imagined the rotation as a consequence of their own hand action. By contrast, lesions in the left M1 sparing the hand area did not lead to an MR deficit (Tomasino et al., 2011), which indicate that the involvement of the hand area of the left M1 cortex is strategy-driven, and that the left M1 supports the motor strategy. That activity in the M1 cortex can be suppressed during tasks tapping motor imagery is not surprising (Solodkin et al., 2004). It has been shown that inputs to M1 are suppressed during kinaesthetic imagery, suggesting the existence of a physiological mechanism whereby the motor system prevents overt movements (Solodkin et al., 2004). Other authors argued that the lack of activation in M1 during motor imagery in their task was caused by the suppression exerted by the SMA emphasizing the role of this region in suppressing movements that are represented in the motor system but not to be performed (Kasess et al., 2008).

## Conclusion

The main points tested by using a quantitative meta-analytic approach (as reported more extensively in the introduction) were:

1. Can the bottom up modulation shed light on MR-related hemispheric lateralization issue? Our results showed that bodily- (vs. non-bodily) stimuli activate a bilateral sensorimotor network, whereas non-bodily (vs. bodily)-related stimuli) included exclusively right-lateralized activations.
2. Can the top down modulation shed light on the debate about whether the sensorimotor cortex is involved in MR? Our results showed that when we directly compared the motor to the visual strategy, we found bilateral activations in the sensorimotor areas.
3. May the M1 activation depend upon the type of stimuli and strategy used? Our results showed that M1 was activated for motor (vs. visual) strategy and bodily- (vs. non-bodily) stimuli.

### Conflict of interest statement

The authors declare that the research was conducted in the absence of any commercial or financial relationships that could be construed as a potential conflict of interest.

## Footnotes

^1^It is known that an additional factor influencing MR mechanisms might be the reference frame (Zacks et al., 2003).

^2^Another account holds that it is the type of judgment that leads to the use of a specific strategy, e.g., (Steggemann et al., 2011).

^3^In behavioral studies it is assumed that this variable can affect MR mechanisms. Two images are presented simultaneously side-by-side and participants are required to perform a same-different decision by judging whether the right stimulus is the same or a mirror-reversed (different) version of the left stimulus. This type of MR may trigger mostly object-based transformation. A body stimulus like a single human figure raising one arm (left or right) presented under different orientations may trigger mostly an egocentric transformation (Steggemann et al., 2011).

^4^Very close to the coordinates derived from (Ehrsson et al., 2003)'s fMRI study on motor imagery and somatotopy of M1 activations: *x* = −8, *y* = −28 and *z* = 64 (left toe/foot) and *x* = 8, *y* = −28 and *z* = 64 (right toe/foot; both in MNI space).
